# Case of Hooping-Cough Attended with Peculiar Symptoms; with Remarks

**Published:** 1857-03

**Authors:** Chas. A. Hentz

**Affiliations:** Marianna, Florida


ART. IV.—*A Case of Hooping-Cough attended with peculiar Symptoms; with Remarks.* By CHAS. A. HENTZ, M.D., of Marianna, Florida.

March 15, 1855.—I was called to see Miss E. B. A——, æt. 11½ years, a well-developed and very intelligent child, whose general health has always been good. Though her mother has always regarded her as delicate, she has never been sick but once, *i. e.* at two years of age, when she had a light attack of measles.

For several days she has been suffering from languor and want of appetite, with constipation of the bowels; and complaining of pain and stiffness in the left side of the neck, behind the mastoid process. She has also had a cough recurring occasionally with considerable violence, but with no hoop, and attended with a moderate mucous expectoration.

The hooping-cough has been prevailing in the family for some time; she has never had it, and has not suffered from any of the initial catarrhal symptoms. The cough just mentioned has existed only a few days.

She remained in bed this morning, and the family were much alarmed to find her in a very strange condition of mental incoherence. She lies uncomplainingly, and sleeps very quietly most of the time; is easily aroused, and looks up with a smile of intelligence, but seems to fail utterly to comprehend the questions asked her. She says "Sir," promptly, when called by name, but to every question she returns the one answer, "It hurts," applying her hand at the same time to the left parietal region.

Her tongue is heavily covered with a thick, smooth, dark yellow coating, and her breath is excessively offensive. Her bowels have been, as I have said, torpid; a dose of calomel, given twelve hours ago, has operated twice, the stools being very offensive, semi-consistent, and very black; her skin is soft, moist, and pleasantly warm; pulse soft and natural in frequency; breathing perfectly quiet and regular.

Not being able to make out a satisfactory diagnosis as to the encephalic disturbance, I prescribed only for the gastric derangement. I gave a dose of calomel, rhubarb, and jalap, which she vomited immediately. This was followed by 8 grs. of calomel, which she retained; and I directed an enema of starch, with 25 drops of spirits of turpentine, to be administered every 4 hours. I also left, for the cough, an expectorant mixture, composed of syr. squills, wine, and ipecac., and sol. sulph. morph.

16th.—Condition unchanged; two alvine evacuations passed during the night, of the same character as those reported yesterday. The cough is ameliorated by the use of the expectorant.

I gave calc. magnesia, ʒss; pulv. rhei, grs. viij; and directed calomel, gr. ij, and ipecac. gr. ss., to be given every two hours; the turpentine enema every six hours.

17th.—In the same condition; bowels moved only as yesterday. I discontinued the calomel, placed a blister over the epigastrium, repeated the cathartic, and continued the expectorant.

18th.—In the same condition. I applied two cups on the left side of the neck, under and behind the mastoid process, and followed them with a blister to the same place; repeated the cathartic, and continued the expectorant.

19th.—The parents report this morning that about 3 o'clock, during the night, she exhibited symptoms of prostration and impending dissolution, her pulse becoming weak and feeble, the surface cool, and breathing gasping and labored. With friction and warmth to the surface, she soon rallied from this condition.

I find her this morning much more dull and languid; there is a very marked dulness or disability of motion about the right side of the body; the tongue is thrust out with difficulty. She still says "it hurts," and applies her hand to the left parietal region; her cough is the same; discharges from the bowels unchanged in character, and only dejected after the admistration of cathartics.

I prescribed strychnia, gr. j; alcohol, ʒiij; sp. lav. comp. ʒj; and directed a dose to be given every three hours, beginning with 5 drops, and increasing one drop at each dose until 8 are given; the effects to be carefully watched. Finding also the fourth and fifth cervical vertebræ tender on pressure, I extended the blister over this region.

20th.—I was pleased this morning to find a very decided change for the better. The phenomena, reported by the family as occurring during the night, are as follows: after taking the third and fourth doses of the strychnia, she began to complain of soreness over the whole of the right side, and started in sleep whenever touched on that side; the soreness seemed to become general in a few hours, and equally marked on both sides. This was distinctly observed by all the attendants, and reported without any prompting on my part.

The difficulty of motion on the right side and of protruding the tongue has all passed away. She answers correctly all questions put to her, though she seems unwilling to talk much; she asks for water, and has called for her pet kitten; and, most interesting of all, she has had several very violent paroxysms of hooping-cough.

She improved steadily. The cough, for the first few days, was attended with an abundant mucous expectoration, and the hooping was excessively violent. The evacuations from the bowels became healthy in appearance, and the tongue cleaned entirely by the 22d. The strychnia was continued for a few days, in the dose of 8 drops every 4 hours.

On the 23d, her parents discovered accidentally that she had forgotten how to read. Though she read very fluently before her illness, she now spelt words slowly and with difficulty, and pronounced and connected them as lamely as if she were just beginning to learn. As she recovered her health, however, her knowledge of reading returned gradually and spontaneously; and from the date of her recovery, a new impetus seemed given to her growth and development.

The phenomena presented by the above case are exceedingly interesting, and seem to me to throw much light upon the mooted subject of the pathology of hooping-cough. Without enumerating the different theories that have been advanced upon this subject, it will be sufficient to observe that the pathological condition clearly indicated in the symptoms of this case is precisely the reverse of that which is supposed in the theory advanced by Desruelles, and held by many, *i. e.* that the disease is at first inflammatory and afterwards spasmodic; that bronchitis is always primitive; cerebral irritation consecutive.

In this case the disease certainly exhibited itself as a cerebral irritation primarily; functional disturbance in the medulla oblongata and cerebral hemisphere on the left side, manifesting itself in the symptoms described, viz., pain and stiffness in the left side of the neck; pain in same side of the head; disturbance of the mental faculties; torpor of the liver and of the gastric function; and, at a more advanced stage, dulness of motion on the right side of the body, and increasing general disability. The normal development of this irritation would have been in the establishment of an excentric irritation in the distribution of the pneumogastric nerve, in the form of hooping-cough; but, from some unknown condition of the system, this was hindered, and the consequence was the above train of concentric phenomena.

The nervous lesion, whatever be its nature, that constitutes the *ipsissimus morbus* of pertussis, is such as, when not embarrassed or complicated by any other abnormal condition that the system may be in, finds a perfect relief in the establishment of the spasmodic cough. In this case the coincidence of complete and immediate relief of the cerebral symptoms on the establishment of the paroxysms of cough on the morning of the 20th, is most marked and decided—a free and sudden unlocking, as it were, of the barrier that was restraining the legitimate action of the *vis conservatrix*, and complete unmasking of the disease.

The arousing of sensibility on the right side of the body when the nervous energy was thus being awakened, is a very interesting and suggestive phenomena.

The connection between the administration of the strychnia and the establishment of these phenomena, is another highly interesting feature of the case. I had been induced to prescribe it on the preceding day rather empirically, in the hope that the nervous system might be aroused by the roborant qualities of the medicine, from the state of torpor and inaction into which it seemed sunk. The result certainly surpassed my expectations.

